# The challenges and opportunities of major trauma care in Germany – results of multidisciplinary expert interviews of the LeAf trauma study

**DOI:** 10.1007/s00068-026-03350-x

**Published:** 2026-10-07

**Authors:** Anne Neubert, Sebastian Hempe, Dan Bieler, Carina Jaekel, Catharina Gaeth, Christine Höfer, Stefanie Bartha, Elisabeth Schwojer, Rolf Lefering, Gina Royko, Denise Schulz, LeAf Trauma study group, Daniela Rodrigues Recchia, Jan Koetsenruijter, Lars Becker, Lisa Wienhoefer, Marcel Dudda, Wiebke Schirrmeister, Susanne Drynda, Felix Walcher, Claudia Heilmann, Patrik Droege, Christian Guenster, Katharina Fetz

**Affiliations:** 1 Department of Orthopedics and Traumatology, University Hospital and Medical Faculty, Heinrich-Heine-University Duesseldorf, Duesseldorf, Germany; 2 Department of Orthopedics and Trauma Surgery, Reconstructive Surgery, Hand Surgery, Plastic Surgery and Burn Medicine, German Armed Forces Central Hospital, Koblenz, Germany; 3 AUC - Academy for Traumatology @ German Society of Traumatology, Munich, Germany; 4 Institute for Research in Operative Medicine, Witten/Herdecke University, Cologne, Germany; 5 University Clinic for Trauma Surgery, Otto von Guericke University Magdeburg, Magdeburg, Germany; 6 Chair of Research Methodology and Statistics, Witten/Herdecke University, Witten, Germany; 7 University Hospital and Medical Faculty, Heinrich-Heine-University Duesseldorf, Duesseldorf, Germany

**Keywords:** Major trauma, Return to work, Polytrauma, Expert interviews, Qualitative research

## Abstract

**Background:**

The recovery process of major trauma survivors involves various sectors within a complex system of different medical service providers and stakeholders who accompany and support patients throughout the recovery pathway. The aim was to explore experiences and opinions of experts, who are actively involved in the care of major trauma patients in the German healthcare system.

**Methods:**

Semistructured in-depth group interviews were used to investigate experiences and opinions of experts with the focus on exploration of expert perspectives on opportunities and challenges of the cross sectoral care. An exploratory analysis was performed with an artificial intelligence-based coding software. In a second step, a system map was developed, and all inter- and intra-sectoral challenges and all opportunities for the optimization of processes were mapped.

**Results:**

39 experts from 13 medical specialties and occupational groups who were interviewed highlighted that intersectional issues especially across and within sectors pose a significant challenge to patient care. Four challenges were highlighted that significantly influence patient care: (1) Communication between sectors; (2) Complexity of transitions between sectors; (3) Inadequate aftercare and (4) Lack of psychosocial care.

**Discussion:**

In conclusion five key learnings can be derived which could help to transform the healthcare system towards a system in which the complex condition major trauma with its high potential of chronification is enabling patients towards living a self-determined life and is creating opportunities for patients to return to work: (1) interdisciplinary collaboration (2), improved communication (3), early psychosocial screening and intervention (4), continuity of care (5), involvement of relatives.

**Supplementary Information:**

The online version contains supplementary material available at https://doi.org/10.1007/s00068-026-03350-x .

Manuscript.

## Introduction

The survival rate of patients after major trauma has significantly improved in recent years. Consequently, the focus has increasingly shifted from mortality to long-term functional and psychological outcomes, reintegration into working and social life, as well as health-related quality of life (hrQoL) [1]. The recovery process of major trauma survivors involves various sectors within a complex system of different medical service providers and stakeholders [2]. These include amongst others, diverse medical professions, social workers, and insurance providers, who accompany and support patients throughout the recovery pathway, from acute care and rehabilitation to professional and social reintegration, all aiming to ensure an optimal and patient-centered recovery.

To examine aspects of recovery of major trauma survivors in relation to return to work and hrQoL, the large-scale multicenter study LeAf Trauma (“Lebensqualität und Arbeitsfähigkeit nach schwerem Trauma”) was conducted in Germany [3, 4]. The previously published qualitative analysis “Lived experiences of working-age polytrauma patients in Germany - A qualitative Analysis” analyzed the experiences of major trauma survivors. Highlighting dissatisfaction with the German healthcare system, the crucial roles of social support by family and friends and the physical and psychological burdens following trauma. In addition, it identified communication barriers between healthcare professional and a need for continuous support via a central point of contact [5].

Major trauma care is complex. Understanding this highly complex system—with its various sectors and processes—can help improve the system and enhance patient outcomes [6]. While the patient’s perspective on the recovery process has been examined, the viewpoints of health professionals involved in the recovery process of major trauma survivors, along with their perspective on common challenges and opportunities for improvement remain underexplored. To further investigate these aspects, gain a realistic impression, and reflect on common challenges in the recovery of major trauma patients, it is essential to include these expert opinions.

Therefore, this study aims to explore experiences and opinions of experts, who are actively involved in the care of major trauma patients in the German healthcare system using semi-structured exploratory group interviews.

## Methods

**Fig. 1:**
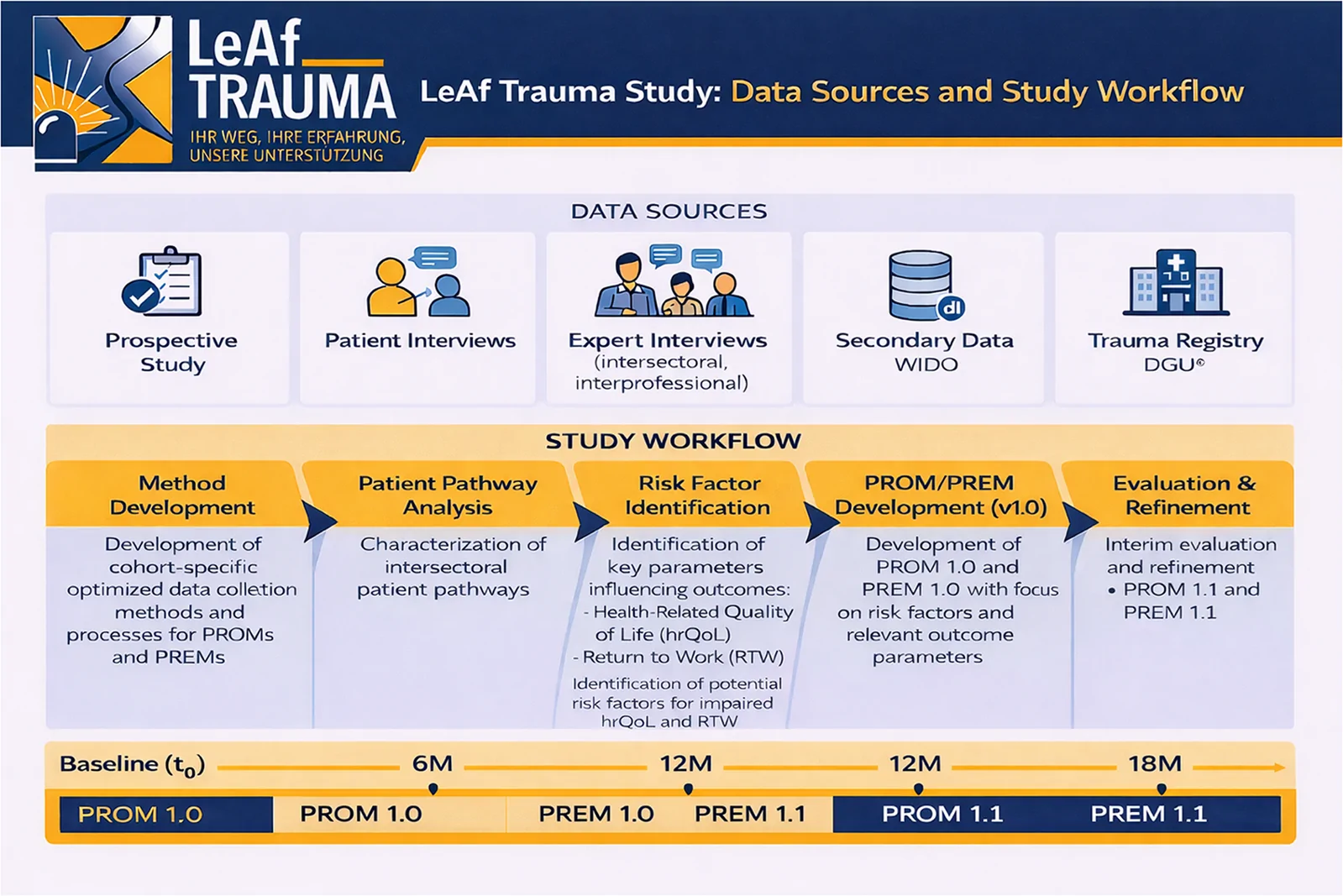
LeAf trauma study - data sources and study workflow – chart was designed with ChatGPT Go

The expert interviews are part of the German major trauma study - LeAf Trauma funded by the Innovation Fund of the Joint Federal Committee Germany (funding reference number 01VSF21033) and registered on the 25 October 2022 under the clinical trial registration number DRKS00028841. For the outline of the entire study with its several study arms (qualitative, retrospective analysis of electronic health data and prospective cohort study – visualized in Fig. 1), please refer to the study protocol by Fetz and colleagues 2024 [3, 4].

### Study design

The study focused on the identification of challenges and opportunities in the cross-sectoral care of major trauma patients with an exploration of expert perspectives. For this purpose, semistructured in-depth group interviews were used. A positive vote from the ethic committee of the Heinrich-Heine-University Düsseldorf was obtained (study ID: 2022 − 1970).

### Participants and recruitment

Within the LeAf Trauma study group, we collected a list of (medical) disciplines involved in the care of major trauma patients through the entire healthcare system (from pre-hospital to ambulatory care – encompassing the entire recovery pathway). These (medical) disciplines were than matched with the respective German Medical Societies (“Fachgesellschaften”) or professional organizations (e.g., “Berufsverband“). A full list of societies and professional organizations invited can be found in the supplementary material 1. All were invited via email requesting the designation of a representative and a substitute for expert group interviews. The invitation letter included general information on the LeAf Trauma study and specific information on the interviews. Up to two reminders were sent over a time frame of two months where necessary.

Further, within the LeAf Trauma study group a list of known experts from orthopedics, trauma and rehabilitation involved in major trauma care in Germany was collected. Together with the designated experts from medical societies and professional organizations, all experts were invited via email. Up to two reminders were sent to the experts over a time frame of one month where necessary. Prior to the interviews all experts were asked to provide written informed consent. The experts were informed a priori about a financial compensation of 100€/per hour for their participation. The financial compensation was financed by the project funds. The interviews were scheduled to last two hours. All interviews were recorded.

### Description planning and conduct of interviews

Semistructured interviews were used to enable discussion among the interviewed experts. The interviewer used a flexible protocol with open questions and additional follow-up questions which could be used to foster discussion [7, 8]. The interviews were conducted online in interprofessional groups of three to seven experts in Juli and September 2022. The interviews content was structured in three blocks: (1) patient pathways, (2) ideas for optimization of healthcare processes and structures, and (3) work ability & hrQoL.

### Analysis

A word document with a verbatim transcript of all interviews was made. During transcription all person-identifying features were anonymized. An exploratory analysis was performed with the artificial intelligence-based coding (AI coding) software ATLAS.ti 2 [9]. ATLAS.ti is a computer-assisted qualitative data analysis software which is offering also an AI-driven co-pilot platform which is powered by a partnership with OpenAI (GPT models). Hence, AI was used for pre-coding of the theme to derive an overview of topics discussed by the experts. For this purpose, intentional AI coding was used in which the authors provided the research intent, research questions and goals. The software then directly coded categories based on the author’s input. These categories were used to manually explore the challenges, opportunities and ideas discussed by the interviewed experts. For all further steps, no AI software was used. With expertise from the Leaf Trauma study team, a system map of the whole intersectoral major trauma treatment path was developed. The original system map can be found in the supplementary material 2. All intra- and intersectoral challenges were mapped manually by the authors. Following this, all opportunities to tackle these challenges as well as ideas for the optimization of processes were integrated in the system map. These helped to explore the complexities, overlaps and interconnections.

## Results

In seven group interviews a total of 39 experts took part, 17 of which were nominated by 13 medical specialties and occupational groups. Experts represent ten German medical societies and professional organizations:

- German Trauma Society (DGU),
- German Society for Orthopedics and Orthopedic Surgery (DGOOC),
- German Society for Orthopedics and trauma (DGOU),
- German Society for Physical Medicine and Rehabilitation Medicine (DGPMR),
- German Pain Society,
- German Society of Neurosurgery (DGNC),
- German Society for Interdisciplinary Emergency and Acute Medicine (DGINA),
- Clinical Social Work Section of the German Association for Social Work,
- Federal Association of Occupational Therapists in Germany (BED), and
- German Society for Physiotherapy Science.

Some of the experts had a mixed occupational group affiliation. In three cases this concerned the specialties orthopedics & trauma, rehabilitation & emergency medicine and in one case orthopedics & trauma and accident insurance (Table 1).

**Table 1:** Participating medical specialties and occupational groups

Medical specialty / Occupational group	Number of persons
Orthopedics & trauma	11 - 10 from inpatient sector - 1 from outpatient sector
Physiotherapy/Ergotherapy	8 - 6 from inpatient sector - 2 from outpatient sector
Rehabilitation/Physical Medicine	5
Anesthesia & Pain medicine	3
Accident insurance	2 - 1 doctor - 1 accident investigator
Rehabilitation counselor/manager	2 - 1 occupational accident insurance clinics - 1 liability insurance
Social worker	2
Emergency physician	1
General practitioner	1
General & visceral surgery	1
Neurosurgery	1
Nursing	1
Orthopedic technician	1
	**39**

Overall, the interviews show that intersectoral issues especially across and within sectors pose a significant challenge to major trauma care. The experts highlighted four challenges that significantly influence patient care. In the following, all challenges will be outlined and accompanied by possible opportunities for change as discussed by the experts.

### Challenge 1: Communication between sectors

Insufficient or a lack of communication between disciplines and/or professional groups (various actors including physicians, therapists, and insurances) was discussed. These communication issues can lead to misunderstandings, important information not being exchanged in a timely manner and a loss of information which is impairing patient care.

It was emphasized that communication between healthcare sectors especially between acute care and rehabilitation, but also between rehabilitation and ambulatory/outpatient sector is often inadequate. After discharge from the acute care hospital, patients often lose contact with their treating physicians, resulting in inadequate aftercare. One expert summarized it like this:

[…] this phrase, ‘interprofessional collaboration in healthcare’ is often just a phrase and isn’t really put into practice, and unfortunately, we still have this—I’d say inadequate—division of responsibilities, as well as a lack of communication on an equal footing, in my view […].

Additionally, the quality of information transfer e.g., by means of documentation or discharge papers was by some of the experts described as inadequate. According to the experts, patients often arrive at rehabilitation clinics with incomplete discharge papers, which is delaying treatment. It has been reported that information regarding medications or imaging findings are frequently unavailable, making it difficult to plan necessary treatments and to ensure the continuity of care. One experts pointed out that:

I can pull out dozens of discharge papers from hospitals in the transfer situation to us [rehabilitation], where it is not clear how much weight-bearing he [the patient] is allowed. Because we often don’t get the discharge papers at all. We have the patient standing here [in the rehabilitation], we have no [radiological] images, no discharge papers, so this is a systemic problem, but I don’t want to point the finger at anyone. Because I say, okay, it’s not because they don’t want to, but because it’s often a problem that you just can’t do it.

#### Opportunities

Hence, better communication between the various actors in the healthcare system, including insurances, was considered by the experts as one necessary step to optimize care. The experts discussed interprofessional case conferences and joint training sessions as an opportunity to improve communication and information delivery between various actors within the acute hospital. One expert summed up the opportunities resulting from interprofessional treatment approach e.g. by case conferences:

I believe that the reality is that when you address the patient on multiple levels—including, of course, the physical, psychological, and emotional aspects […] with a well-coordinated, interdisciplinary therapeutic program, when you approach the patient or the injured person in this way, you certainly generate a significantly higher level of intrinsic motivation than if you just let the patient therapeutically drifting along. If the patient has a very clear direction, a very clear path, with defined goals […] I believe that, in this respect, the interdisciplinary approach with a well-structured therapeutic plan certainly boosts motivation.

It was proposed that therapists (e.g., physical therapists, occupational therapists) also have access to the physician’s notes and be given the opportunity to contribute their reports to the medical records. This could promote interprofessional communication and ensure that all relevant information for the patient’s treatment is available. Greater interprofessional collaboration between different departments and actors in the acute hospital could ensure a more holistic care of major trauma survivors. However, also beyond the acute hospital the experts recommended that communication needs to be improved e.g., between acute care hospitals and rehabilitation facilities to ensure that all necessary information is shared in a timely manner. This could be fostered by introducing/expanding/using of digital communication tools. One expert pointed out that there would be a need for ongoing training for medical staff on the importance of documentation and the proper preparation of discharge papers. Further, modern systems exist for the (semi-)automated generation of discharge papers that include important information such as medication lists and lab results. Both training and automation could enhance the quality of documentation and ensure that all relevant information is provided quickly and efficiently.

Further experts discussed forms of feedback to continuously improve the quality of documentation and to ensure that all parties involved are kept informed of the patient’s progress. One expert resumed:

I think I might describe it in two words: continuity and feedback. So, we should have as few people as possible involved in this optimal process. […] but a feedback mechanism needs to be built in feedback after the so-called emergency phase, feedback after the first phase, and feedback after the second phase during inpatient care. It’s difficult, but I think continuity and feedback are the two words I’d like to throw out there.

### Challenge 2: Complexity of transitions between sectors

Transitions between different sectors of care (e.g., from acute hospital to rehabilitation) are often complex and thereby causing it to be poorly organized. Major trauma survivors pass through many intersections, which can lead to confusion and delays in treatment. Further, variations in standards and procedures across different facilities can lead to inconsistencies in patient care, which complicates transitions between the various sectors. One expert described it like this:

We use the same medical care for all patients—though we have different regulations and different mandates—but these procedures are the only ones that vary. This creates so much friction because they aren’t standardized; instead, I have to check: Is this a patient covered by statutory health insurance? Which health insurance plan is it? What are the regulations by this statutory health insurance for this patient? For some, the health insurance provider determines the follow-up care facility or rehabilitation program. For others, it doesn’t, so the patient can be registered directly—and that’s an extremely time-consuming, resource-draining process where I think to myself, ‘Actually, the colleagues in the hospitals and the social services should have something else to do‘.

Several of the experts discussed challenges in relation to the so-called rehabilitation gap, describing that many major trauma survivors are not directly discharged in a rehabilitation facility following the acute hospital stay but rather discharged home with or without physical therapy. Further, the experts highlighted the so-called aftercare gap, in which major trauma survivors do not receive adequate aftercare e.g., psychotherapy and/or physical therapy to further improve physical, social and psychological well-being in order to return to work and to enjoy individually defined hrQoL. One expert summarized this challenge like this:

Accordingly, he [the patient] is then classified as eligible for discharge from the acute care facility, but not yet ready for rehabilitation, so that he [the patient] falls into the [rehabilitation] gap between these two forms of care and is then transferred to a short-term care facility or, even more dramatically, sent home, where basic care measures are then provided, sometimes in the form of nursing care or, at best, physiotherapy with home visits.

The experts reported different disease concepts and treatment approaches between the various disciplines. This leads to misunderstandings and difficulties in interprofessional collaboration, which impairs the continuity of care as one expert outlined:

[…] the focus on illness varies greatly between disciplines, I believe. And that’s why it’s sometimes difficult to understand each other. Yes, because there is often no uniform concept of illness. On the one hand, there is perhaps a very mechanistic concept of illness, where something is broken and now has to be healed, shut down, or whatever. And then on the other hand, there is perhaps a focus on stress factors or similar things. I think that’s a very important part, and the inner hospital interfaces are already a problem.

This situation is further exacerbated by bureaucratic hurdles patients face during their recovery, particularly those related to cost bearers and approvals. The experts pointed out that applying for rehabilitation services is perceived as bureaucratic and complicated especially in relation to patients treated outside the context of the statutory accident insurance hospitals (BG Hospital). Patients often have difficulties submitting the necessary applications to (health) insurances, which leads to frustration and delays. However, another expert said that:

In my experience, this isn’t really a bureaucratic problem, but rather a resource problem. We can’t get our patients discharged simply because the rehabilitation clinics don’t have the capacity to admit them. […] But now I’m sitting here on my high horse because we have a case management team, and I tell them he needs to go to rehab, and they handle the application, […] The fact that you have to submit an application, that they also have to get to know the patient—to be honest, I actually think the bureaucratic hurdles are super low, almost dangerously low.

Further, experts referred to communication issues as described in Challenge 1. Inadequate communication between the various players in the healthcare system, including acute care hospitals and rehabilitation facilities, exacerbates this challenge and can lead to important information being lost and patient care being compromised.

#### Opportunities

The experts discussed several forms of rehabilitation counselors/managers or case managers for all patients regardless of the cost bearer but in the form of the rehabilitation management which is customary at BG Hospitals. The experts’ opinions about rehabilitation managers were predominantly positive. Many experts saw rehabilitation managers as very useful and helpful, especially in supporting patients through the bureaucratic jungle of rehabilitation. One expert described rehabilitation manager in the setting of the BG Hospitals as *“the best invention they’ve had in the last 15 years”* and emphasized that it is an enormous help for major trauma survivors. However, it was also pointed out that cost pressure and bureaucratic hurdles can impair the effectiveness of rehabilitation management. One expert noted that cost pressures make it difficult to provide the necessary resources to improve rehabilitation processes. The person stated:

Yes, it [the rehabilitation manager] is like a family doctor. You go to your family doctor and talk about it, and then you feel heard, understood, and acknowledged. And that’s exactly what can ultimately give patients an incredible boost. It’s much better than someone saying something about reintegration three months ago. Instead, you’ve decided on it and agreed on it. […]. This means that ultimately, communication with the person concerned, with the rehabilitee, takes place in a way where you don’t decide something, but where you agree on something. And if you take that into account, it has a great success rate. But of course, it would be nice if this could happen not only for people injured in work accidents, but also for all other statutory insured persons.

Interdisciplinary collaboration between the rehabilitation manager, medical disciplines, and therapists was stressed as very important. It was argued that close communication and collaboration between these various actors is crucial for the success of rehabilitation of major trauma survivors. Further, the experts stressed that training and wide knowledge of the different legal systems, specific care structures and patient needs is essential to adequately address the complex needs of major trauma survivors. With such a rehabilitation manager at hand, early planning of the rehabilitation while the patient is still in the acute care hospital could be achieved. However, this would also require the availability of appropriate rehabilitation facilities that are tailored to these specific needs.

### Challenge 3: Inadequate aftercare

After discharge from rehabilitation, there is often no defined aftercare concept established, especially for patients with statutory health insurance as the experts discussed. While patients after work-related accidents have a rehabilitation manager and an accident insurance doctor *[“Durchgangs-Arzt”]* who takes care of their needs also beyond inpatient rehabilitation, such a point of contact is only rarely existing for patients covered by statutory health insurance. General practitioners are often not informed that a patient has been e.g., in intensive care, which means that aftercare is not adequately continued, not tailored to their needs. Causing patients who do not take the initiative themselves drop out of treatment. Hence, many patients fall into a care gap after rehabilitation (*‘aftercare gap’*), as due to a lack of a clear aftercare concept, leading to a long period without adequate support regarding e.g., general treatment, physical/ergotherapy and psychological treatment. One expert described that:

[…] even in the case of ‘normal patients’ [referring to a non-major trauma patient], it can happen that after rehabilitation, there is simply no one responsible for ensuring that the patient continues to receive treatment. The general practitioner is not aware of this, cannot contact the patient, and if the patient does not take the initiative themselves, then the treatment stops, or rather, it discontinues.

Experts stated the lack of a defined point of contact to coordinate aftercare for major trauma survivors. This is especially the case for care situations outside the BG Hospital context. Hence, patients slip into an aftercare gap - not receiving the support and care needed. Establishing a point of contact would therefore be beneficial. One expert explained one of the issues of aftercare:

[…] Then there’s the issue with medications: sometimes the acute episode happened so long ago that those six months of budget-free prescriptions without an economic feasibility review are no longer an option, so that the problem arises again where [outpatient] practitioners have to watch their budgets, and then, in some cases, with extremely limited care or even virtually no care at all, the case is closed with the statement, ‘There’s nothing more we can do for you.’ So, this is precisely the part that is sometimes truly dramatic, because despite visible, recognizable major deficits, in some cases no further physical therapy or occupational therapy measures are prescribed. In my view, this highlights the two biggest interface issues between acute care hospitals and inpatient facilities, as well as between inpatient rehabilitation and outpatient follow-up care.

#### Opportunities

The experts emphasized that many patients require long-term support to maintain their hrQoL and facilitate their return to work. This requires structured aftercare that goes beyond immediate rehabilitation. They pinpointed that a designated point of contact to coordinate aftercare e.g., in form of a “polytrauma outpatient service” within the acute care hospital would be essential to ensure that patients receive the support needed throughout the entire recovery process without slipping into an aftercare gap. This point of care in combination with the rehabilitation manager could also improve patient navigation through the complex healthcare system (Challenge 2). Further, the importance of family involvement to maximize the support was discussed.

### Challenge 4: Lack of psychosocial care

Psychosocial factors are not sufficiently recognized, the experts agreed. Health care professionals need to be made more aware of this issue in order to be enabled to better identify and address patients’ psychosocial needs. One expert pointed out that:

For example, a patient who has suffered some kind of trauma and doesn’t know whether he will be able to keep his job, support his family, pay off his mortgage, and so on. These are things that have a direct impact on the psychosocial side of acute treatment. And unfortunately, we often find that these aspects are not initially recognized or do not play a relevant role in a somatic context of trauma surgery, where the focus is naturally quite different. We don’t have any structure for this in the hospitals.

Additionally, it was discussed that the response to psychological distress varies greatly from person to person. Some patients may require more intensive support than others, which calls for a tailored approach. However, it was noted that there are not enough qualified professionals to meet the demand which causes considerable waiting times for psychotherapeutic treatment. As a result, many patients do not receive the necessary support. This worsens the mental health of major trauma survivors which also makes it more difficult for them to return to work.

#### Opportunities

Experts highlighted the opportunity in mental health screening and early supportive interventions. It was recommended that psychosocial screening, care and rehabilitation shall begin as early as possible, already in acute care as it can significantly improve patients’ chances of recovery. Intervention shall focus on resources as much as deficits in order to motivate patients and increase self-efficacy. One expert put it like this:

If we were to take that as a basic premise—saying that every injury has a psychological component just as it has a physical one—then, if we follow that line of reasoning to its logical conclusion, we would actually have to make this therapeutic option mandatory for the patient, at the very least. We don’t ask patients if we can operate on them—well, of course we ask them according to standard practice, but patients who are injured, who have injuries requiring surgery, are operated on. Right? And if we assume that they will inevitably suffer psychological damage in the process, then we would probably have to not only offer them these therapies in the end, but—as part of a screening—even provide them in the patient’s best interest; I don’t want to say force them but certainly make them available.

Further, the involvement of the family members was discussed to be benefitable. Psychosocial support should extend not only to patients but also to their family members. Family members play an important role in supporting patients and should therefore be involved in the rehabilitation process. According to the experts, also informational sessions and training of family members would be very beneficial to strengthen their role in the recovery process. An expert described the importance of psychosocial support and family involvement like this:

[…] this is a very important issue that should be handled by an experienced psychologist in this field. But I think from the patient’s perspective, it’s also a bit of a balancing act because, at the beginning—as a polytrauma patient—they have a high need for acute medical care. […] And then early rehabilitation—the need for early rehab—slowly starts to kick in as acute medical diagnosis and treatment take a back seat. Then you have to figure out when you might be able to diagnose whether the patient has this [psychological/psychiatric/psychosomatic] condition. And at what point can I start treating them [the patients]—and I also have to be careful not to overwhelm them [the patients] by drawing too heavily on their own resources. And there’s the issue of rehabilitation readiness and treatment readiness again. I think you have to be careful about when you start working with the patient. I think this is a long, drawn-out therapy […] and then, of course, you have to consider again whether the closest relatives, spouse, or partner should be involved or not.

## Discussion

Through the expert interviews, it was possible to discover four main challenges and the opportunities the experts described to counteract or even overcome these challenges. The results echo those of the literature and other findings from the LeAf Trauma study such as the patient interviews [5]. The four challenges share a common agreement to better integrate family members into the recovery processes. This could make treatment more holistic, as well as it can help to better integrate and prepare both families and patients for the long recovery processes.

Issues in relation to communication and documentation were discussed by the experts in the current study but resemble also findings within the patients interviews of our LeAf Trauma study group as well as other studies in the field [5, 10]. In the current study mostly interprofessional communication was stated as problematic whereas patients stated interprofessional as well as patient communication issues [5]. This is in line with what Joeng (2024) points out in his editorial. Major trauma care is characterized by its multiprofessional teams necessary to effectively address the needs of patients. Each specialist involved plays an essential role in the treatment and recovery process. Essential for the efficacy of these multidisciplinary teams is effective coordination and communication which could be fostered by regular meetings and comprehensive documentation as well as joint decision making [11].

As such a “polytrauma board” were those involved in the treatment of major trauma patients come together to design an individual treatment plan would be beneficial. Kaske and colleagues (2014) argue that such multiprofessional meetings are already the norm for other diseases such as in cancer treatment (e.g. tumor boards) but not so in major trauma care. Even though, these patients suffer a broad range of physical, mental and social impairments which requires a multiprofessional team of specialists who address these needs [12]. A “polytrauma board” could, hence, improve interprofessional communication, treatment and outcome of patients [13, 14]. Lugeder and colleagues (2013) stress that communication is of paramount importance. For them multiprofessional team meetings form the key for e.g., early identification of complications and the development of solutions which can help to foster an individual and holistic recovery process [15].

Further, with a wide range of information needed to ensure the continuity of care of major trauma patients, digitalization of health data is a great opportunity to enhance the quality of documentation and information delivery across sectors. In general, studies state that the digitalization within the healthcare system can achieve three broad outcomes: improved patient outcomes, operational efficiency, and increased patient engagement [16]. All of which could significantly enhance the care of major trauma survivors and contribute to foster care work across sectors within a complex system with various actors including multiple different cost bearers.

Nonetheless, even with sufficient digitalization of health data across sectors, the system will remain complex due to various sectors with intersecting processes which link across local and regional infrastructures and services [17]. Hence, it needs experts within the system who help patients navigate. In Germany the concept of a rehabilitation management is well established in the context of BG Hospitals. In this system, the rehabilitation manager serves as an active supervisor of the rehabilitation process with the aim to achieve permanent and timely return to work especially in complicated cases [15]. As the experts pointed out, implementing the concept of rehabilitation management across all cost bearers would be an efficient way to ensure individual needs-based approaches to recovery and foster return to work.

Moreover, also with the support of a point of contact/contact person as a navigator of the recovery process, the patient could be enabled to overcome complexities within the healthcare system or at least this contact person could ease the navigation. This would, as pointed out by the experts, help to overcome/ease problems such as the aftercare gap.

This resembles the findings from the patient interviews conducted also as part of the LeAf Trauma study, in which the patients expressed a great need for a continuous contact person involved throughout the entire treatment and recovery process. The interviewed patients pointed out that a general practitioner cannot take on this role due to the complexity of condition and the wide range of residual impairments [5]. Spering and colleagues [17] argue that such a point of contact could be achieved via an outpatient center within the acute care hospital in which the patient was treated. This outpatient center could act as a coordinator of the individual needs-based care of major trauma survivors [18]. Hence, it is essential to ensure continuity of care throughout the entire recovery process.

In this relation also the need for psychosocial care and the lack within the healthcare system was one of the dominant topics within the expert interviews. Further also the need for a more holistic care models of major trauma survivors was discussed but also the lack of resources for psychological support within the healthcare system. Several studies show the impacts of severe injuries on mental health. For example, O’Donnell and colleagues (2004) who show a high prevalence of psychopathologies (mostly frequent in their cohort posttraumatic stress disorder (PTSD) and major depressive disorder at both 3 and 12 months) in the first year after trauma. According to the authors this indicates the high frequency and persistency of psychiatric disorders following major trauma. These findings also are in line with other publication on the mental health consequences of major trauma [12, 21, 22].

The experts pointed out the benefits of early mental health screening and supportive interventions but also determined the appropriate timeframe for such a screening and intervention so as not to overwhelm patients. Also, within the LeAf Trauma patient interviews, our study group showed the need for psychosocial support and that a point of contact could ensure earlier identification and treatment of patients in need (5). Also other authors point towards the responsibilities of the healthcare system to adopt early screening approaches and interventions [21]. This is in line with leading guideline such as the NICE guideline on rehabilitation after traumatic injury which state that after major physical trauma, many patients experience acute distress and a need for psychosocial support; however, only a subset will develop a mental disorder requiring formal psychotherapy. Early psychosocial support is nevertheless essential and should include humane, supportive, and practical assistance as well as low-threshold supportive conversations and crisis-oriented interventions. In the acute hospital setting, such care may be provided through consultation-liaison with psychosomatic or mental health services, whereas psychiatric involvement is particularly important in the assessment and management of severe mental health disturbance, including e.g., suicidality or self-harm risk. The NICE guideline points out that structured follow-up care after a traumatic injury should also include the assessment of psychological functioning and repeated evaluation of referral needs so that patients developing trauma-related disorders can be identified early [19].

Although PTSD is a key post-traumatic outcome, depressive and anxiety disorders are common, too and should be considered in follow-up patient pathways. In a multicenter longitudinal study by van der Vlegel, 22% of injured patients showed symptoms of a probable mental disorder 6 months after injury, including anxiety, depression, and PTSD [20]. Additionally, our yet unpublished analysis of the prospective cohort of the LeAf Trauma study indicates that mental health symptoms are relevant to major post-traumatic outcomes, particularly hrQoL and return to work. As trauma-related symptoms may develop or persist over time, repeated screening beyond the acute hospital phase is essential. However, screening should not remain an isolated measure; positive findings need to lead to direct diagnostic assessment in order to detect clinically relevant disorders early and to ensure timely referral to appropriate psychosocial and psychotherapeutic care [23, 24]. An overview of psychological support interventions is presented in a current scoping review by Rutetzki and colleagues (2026) [25].

### Strength and weaknesses

This study was able to present a wide range of expert opinions with 39 experts from the field of major trauma care. Hence, this is one of the largest qualitative studies interviewing German experts of major trauma care ever conducted. The participating experts come from various backgrounds covering the inpatient and outpatient sector, resembling the most important professions involved in the recovery process of major trauma survivors which is increasing the transferability as well as internal validity of the study. However, despite invitations sent out to medical societies including reminder, some occupational groups were not represented (e.g., psychiatry, psychotherapy, pension insurance, health insurances, medical service of health insurance companies, wound management, supplier of medical aids) or were underrepresented (e.g., practicing physicians (surgeons and general practitioners)). Despite the absence of experts from e.g., psychology, psychosomatic, psychiatry and neurology the relevance and the urgent need for more psychosocial care was stressed by the participating experts. These experts views highlight a more holistic biopsychosocial understanding of major trauma with its great chronification potential. Hence, major trauma is more than the number of injuries a patient suffered, but disease with many facets.

While these results cannot be generalized for the entire German healthcare system, it is likely that the experiences of those interviewed reflect on a wide range of situations within the German healthcare system and are not bound to the single hospitals/practices from which the experts come. Some of the experts were proposed by members of the study group. This may have increased certain viewpoints, but the diversity of represented opinions suggests that this probably did not have a particular influence on the results. Nonetheless, a certain responder bias is maybe apparent due to the large number of orthopedic and trauma surgeons interviewed.

The application of AI-based software in qualitative research is still at an early stage of development and may introduce several limitations, including potential bias in coding and difficulties in detecting nuanced or implicit themes, as highlighted in recent studies [26–28]. To minimize the influence of these limitations, a double-coding procedure combined with a system mapping framework was applied. AI-assisted coding was primarily used to obtain an initial overview of the topics raised by the experts. The AI tool functioned as a supportive tool for the exploratory analysis of interview content. Meaningful interpretation, however, required the active involvement of the researcher, who (1) must be highly familiar with the data (2), needs to engage closely with the material in order to detect themes and ideas—particularly subtle ones that the software may overlook—and (3) must interpret these themes, relate them to each other, and integrate them within a coherent analytical framework, as also emphasized by Morgan (2023) [28]. All of which was given for the analysis of the present study.

## Key learnings for policy and practice

**Fig. 2:**
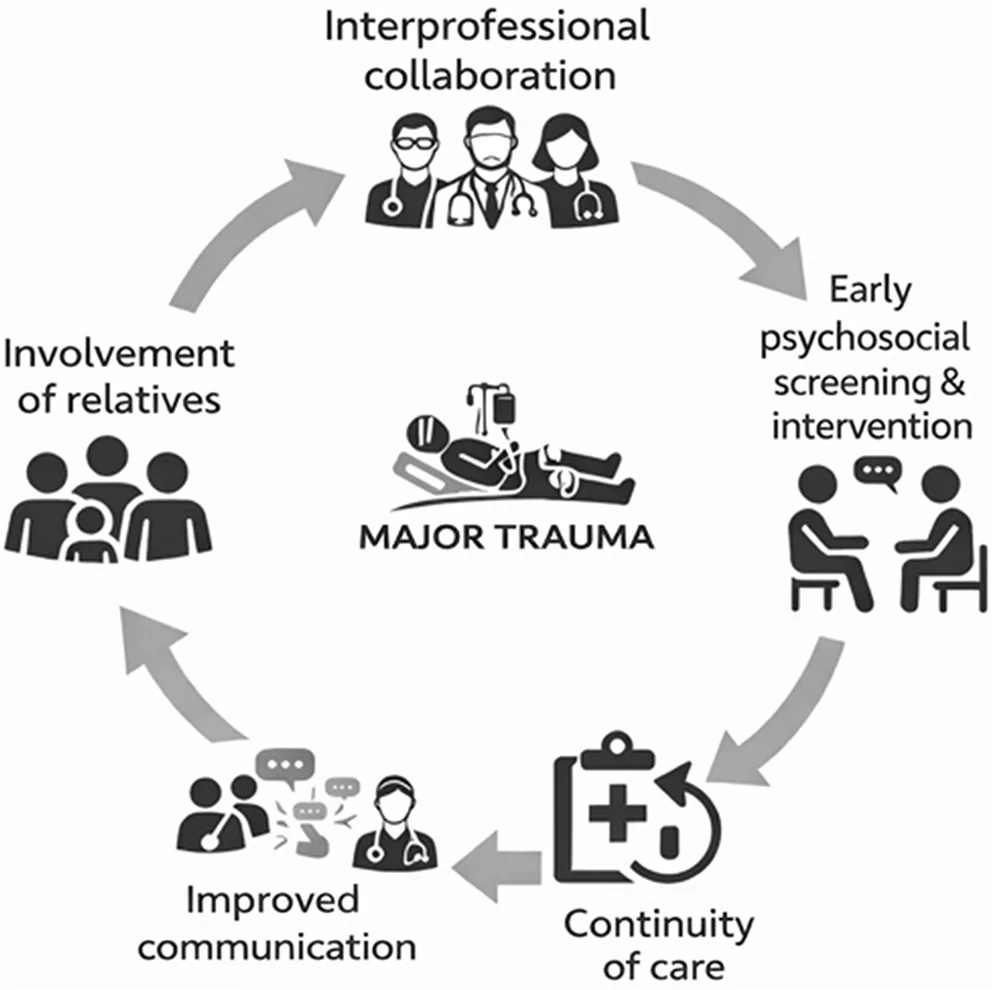
Five key learnings from the expert interviews

In conclusion, five key learnings (Fig. 2) can be derived which could help to transform the healthcare system towards a system in which the complex condition major trauma with its high chronification potential is enabling patients towards living a life worth living on one’s own terms and is creating opportunities for patients to return to work.

1. **Interprofessional collaboration**: Close collaboration between various healthcare professionals is regarded as essential to ensure holistic patient care. This is also promoting communication and the exchange of information. The latter would also benefit from the digitalization of healthcare information.
2. **Early psychosocial screening & intervention**: The early psychosocial screening and intervention, ideally already in the acute care setting, is considered very important – regarding early recovery, chronification, return to work and hrQoL.
3. **Continuity of care**: Ensuring that patients have a designated contact person throughout the entire recovery process is considered essential. This can help to reduce uncertainties, overcome gaps within the healthcare system as well as provide better physical and mental support for patients and family members which will improve patient recovery, hrQoL and fosters return to work.
4. **Improved communication**: Better communication between the various players in the healthcare system, including insurance providers, is considered necessary to reduce bureaucratic hurdles and optimize care.
5. **Involvement of relatives**: The involvement of family members in the recovery process is considered crucial to promote patient support, but moreover it could help that the psychosocial aspects of recovery are better considered.

## Supplementary Information

Below is the link to the electronic supplementary material.

Supplementary Material 1: List of invited medical societies and professional group

Supplementary Material 2: System map – German Trauma Care and Recovery

## Data Availability

The data that support the findings of this study are not publicly available due to restrictions by the data protection law of Germany (DSGVO). These data were only allowed to be investigated under a license for the current study and so are not publicly available.

## Abbreviations

AI: Artifical intelligence
BG: Berufsgenossenschaft (statutory accident insurance)
hrQoL: health-related Quality of Life
LeAf Trauma: Lebensqualität und Arbeitsfähigkeit nach schwerem Trauma
PTSD: Posttraumatic stress disorder

## References

[1] Livingston DH. A fate worse than death? Long-term outcome of trauma patients admitted to the surgical intensive care unit. J Trauma. 2009;67(2):341–9. 10.1097/TA.0b013e3181a5cc34. 19667888

[2] WHO Health Cluster. The Trauma Pathway From A to Z [Internet]. Available from: https://www.emro.who.int/images/stories/palestine/documents/trauma-pathway-4.pdf.

[3] Fetz K, Grimaldi G, Bieler D, Neubert A, Jaekel C, Hoefer C, et al. LeAf Trauma- an intersectoral prospective multicenter study assessing quality of life and return to work after majortrauma-study protocol. PLoS ONE. 2024;19(11):e0312320. 10.1371/journal.pone.0312320 . PubMed PMID: 39536014 ; PubMed Central PMCID: PMC11560036. PMC11560036 39536014

[4] The PLOS One Staff. Correction: LeAf Trauma- an intersectoral prospective multicenter study assessing quality of life and return to work after majortrauma–study protocol. PLoS ONE. 2025;20(9):e0332632. 10.1371/journal.pone.0332632. PMC12440169 40956835

[5] Neubert A, Hempe S, Jaekel C, Gaeth C, Spering C, Fetz K, et al. Lived experiences of working-age polytrauma patients in Germany - A qualitative Analysis. Injury. 2024;111938. 10.1016/j.injury.2024.111938 . PubMed PMID: 39477709 . 39477709

[6] Edmiston T, Bath MF, Caceres E, Nuño-Guzmán CM, Baderhabusha DU, Khajanchi M, et al. Variation in global trauma care: a survey of 187 hospitals across 51 countries. BMJ Glob Health. 2025;10(11):e021784. 10.1136/bmjgh-2025-021784. PMC12598983 41213661

[7] DeJonckheere M, Vaughn LM. Semistructured interviewing in primary care research: a balance of relationship and rigour. Fam Med Community Health. 2019;7(2):e000057. 10.1136/fmch-2018-000057. PMC6910737 32148704

[8] Pope C, Mays N, editors. Qualitative Research in Health Care [Internet]. 1st ed. Wiley; 2006 [cited 2026 Mar 19]. Available from: https://onlinelibrary.wiley.com/doi/book/ doi:10.1002/9780470750841.

[9] ATLAS.ti Scientific Software Development GmbH. Qualitative data analysis software [Internet]. 2023. Available from: https://atlasti.com.

[10] Beckett K, Earthy S, Sleney J, Barnes J, Kellezi B, Barker M, et al. Providing effective trauma care: the potential for service provider views to enhance the quality of care (qualitative study nested within a multicentre longitudinal quantitative study). BMJ Open. 2014;4(7):e005668. 10.1136/bmjopen-2014-005668. PMC4091464 25005598

[11] Jeong TS. A Multidisciplinary Approach to Polytrauma Management. Korean J Neurotrauma. 2024;20(1):1. 10.13004/kjnt.2024.20.e5. PMC10990699 38576497

[12] Kaske S, Lefering R, Trentzsch H, Driessen A, Bouillon B, Maegele M, et al. Quality of life two years after severe trauma: A single centre evaluation. Injury. 2014;45:S100–5. 10.1016/j.injury.2014.08.028. 25284226

[13] Doke S, Karnatapu S, Karnatapu S. The significance of multidisciplinary approach to comprehensive trauma management. Ann Crit Care Emerg Med. 2023;4(1). 10.29011/ACCEM-106.000106.

[14] Ruchholtz S, Waydhas C, Lewan U, Piepenbrink K, Stolke D, Debatin J, et al. A multidisciplinary quality management system for the early treatment of severely injured patients: implementation and results in two trauma centers. Intensive Care Med. 2002;28(10):1395–404. 10.1007/s00134-002-1446-8. 12373463

[15] Lugeder A, Berkenbrink A, Zeichen J. Reha-Management in der gesetzlichen Unfallversicherung: Erste durchgangsärztliche Erfahrungen. Unfallchirurg. 2013;116(3):271–6. 10.1007/s00113-011-2149-9. 22614127

[16] Ebo TO, Clement David-Olawade A, Ebo DM, Egbon E, Olawade DB. Transforming healthcare delivery: A comprehensive review of digital integration, challenges, and best practices in integrated care systems. Digit Eng. 2025;6:100056. 10.1016/j.dte.2025.100056.

[17] Edmiston T, Bath MF, Caceres E, Nuño-Guzmán CM, Baderhabusha DU, Khajanchi M, et al. Variation in global trauma care: a survey of 187 hospitals across 51 countries. BMJ Glob Health. 2025;10(11):e021784. 10.1136/bmjgh-2025-021784. PMC12598983 41213661

[18] Spering C, Bator F, von Steinbüchel N, Neubert A, Awan Malik H, Lehmann W. Beyond surviving: the mental and physical impairment of health-related quality of life after polytrauma emphasizes the need for interprofessional long-term aftercare. Injury. 2026. 10.1016/j.injury.2026.113644. 42721570

[19] NICE. Rehabilitation after traumatic injury NG211 [Guideline] [Internet]. United Kingdom: NICE. 2022. Guideline no.: NG211. Available from: https://www.nice.org.uk/guidance/ng211/resources/rehabilitation-after-traumatic-injury-pdf-66143770162117.

[20] Van Der Vlegel M, Polinder S, Toet H, Panneman MJM, Geraerds AJLM, Haagsma JA. Anxiety, depression and post-traumatic stress symptoms among injury patients and the association with outcome after injury. Eur J Psychotraumatology. 2022;13(1):2023422. 10.1080/20008198.2021.2023422. PMC8803064 35111285

[21] O’Donnell ML, Creamer M, Pattison P, Atkin C. Psychiatric Morbidity Following Injury. Am J Psychiatry. 2004;161(3):507–14. 10.1176/appi.ajp.161.3.507. 14992977

[22] Olive P, Hives L, Ashton A, O’Brien M, Taylor A, Mercer G, et al. Psychological and psychosocial aspects of major trauma care: A survey of current practice across UK and Ireland. Trauma. 2024;26(2):124–33. 10.1177/14604086221145529.

[23] Neubert A, Koetsenruijter J, Rodrigues Recchia D, Bieler D, Schulz D, Bartha S et al. Life after major trauma: predictor of return to work – Results of the prospective LeAf Trauma Study Cohort. Prep.

[24] Fetz K, Neubert A, Rodrigues Recchia D, Koetsenruijter J, Bieler D, Gaeth G, Schulz D et al. Life after major trauma: predictor of health-related quality of life – Results of the prospective LeAf Trauma Study Cohort. Prep.

[25] Rutetzki J, Breuing J, Goossen K, Lucius F, Ostermann T, Fetz K. Psychological care in acute and emergency medicine: a scoping review of support interventions by healthcare professionals. BMC Emerg Med. 2026;26(1):64. 10.1186/s12873-026-01494-y. PMC12930831 41709162

[26] Open AI, How Should AI. Systems Behave, and Who Should Decide [Internet]. 2023. Available from: https://openai.com/index/how-should-ai-systems-behave/.

[27] Friese S. Two case studies on the application of generative AI in qualitative data analysis: Success and disenchantment [social media]. LinkedIn [Internet]. 2023. Available from: https://www.linkedin.com/pulse/two-case-studies-applying-generative-ai-qualitative-data-friese.

[28] Morgan DL. Exploring the Use of Artificial Intelligence for Qualitative Data Analysis: The Case of ChatGPT. Int J Qual Methods. 2023;22:16094069231211248. 10.1177/16094069231211248.

