# Supplementary material for: The challenges and opportunities of major trauma care in Germany – results of multidisciplinary expert interviews of the LeAf trauma study
Source: Eur J Trauma Emerg Surg. 2026 Oct 7;52(1):308. doi: 10.1007/s00068-026-03350-x (PMC13645980; doi:10.1007/s00068-026-03350-x)
Supplement: Supplementary file 1 [file 68_2026_3350_MOESM1_ESM.pdf]

Supplemental file 1: Invited medical societies and professional organisations

- DGU- Deutsche Gesellschaft für Unfallchirurgie
- DGOU - Deutsche Gesellschaft für Orthopädie und Unfallchirurgie
- DEGAM - Deutsche Gesellschaft für Allgemeinmedizin und Familienmedizin
- DGPRM - Deutsche Gesellschaft für Physikalische und Rehabilitative Medizin
- Deutsche Gesellschaft für Endoprothetik
- Deutsche Gesellschaft für interprofessionelle Hilfsmittelversorgung e. V. (DGIHV)
- DeGPT - Deutschsprachige Gesellschaft für Psychotraumatologie
- DGPTW - Deutsche Gesellschaft für Physiotherapiewissenschaft
- alternativ: Deutscher Verband für Physiotherapie (ZVK) e. V.
- Deutsche Gesellschaft für Ergotherapiewissenschaft e.V.
- Bundesverband für Ergotherapeuten in Deutschland e.V.
- Deutsche Gesellschaft für Pflegewissenschaft e.V.
- DGAVC - Deutsche Gesellschaft für Allgemein- und Viszeralchirurgie
- DGAI - Deutsche Gesellschaft für Anästhesiologie und Intensivmedizin
- DGINA e.V.- Deutsche Gesellschaft für interdisziplinäre Notfall- und Akutmedizin
- DGUV - Deutsche gesetzliche Unfallversicherung
- Medizinischer Dienst Bund
- Deutsche Gesellschaft für soziale Arbeit im Gesundheitswesen
- Deutsche Schmerzgesellschaft
- Deutsche Gesellschaft für Wundheilung und
- Deutsche Gesellschaft für Psychiatrie, Psychotherapie, Psychosomatik und Nervenheilkunde
- Deutsche Gesellschaft für Neurochirurgie
- Verband deutscher Rentenversicherungsträger -Dezernat Reha-Wissenschaften:
- Deutscher Hausärzteverband
- Deutsche Gesellschaft für soziale Arbeit (Sektion klinische soziale Arbeit)
- Berufsverband der niedergelassenen Chirurgen
