# Supplementary material for: The challenges and opportunities of major trauma care in Germany – results of multidisciplinary expert interviews of the LeAf trauma study
Source: Eur J Trauma Emerg Surg. 2026 Oct 7;52(1):308. doi: 10.1007/s00068-026-03350-x (PMC13645980; doi:10.1007/s00068-026-03350-x)
Supplement: Supplementary file 2 [file 68_2026_3350_MOESM2_ESM.pdf]

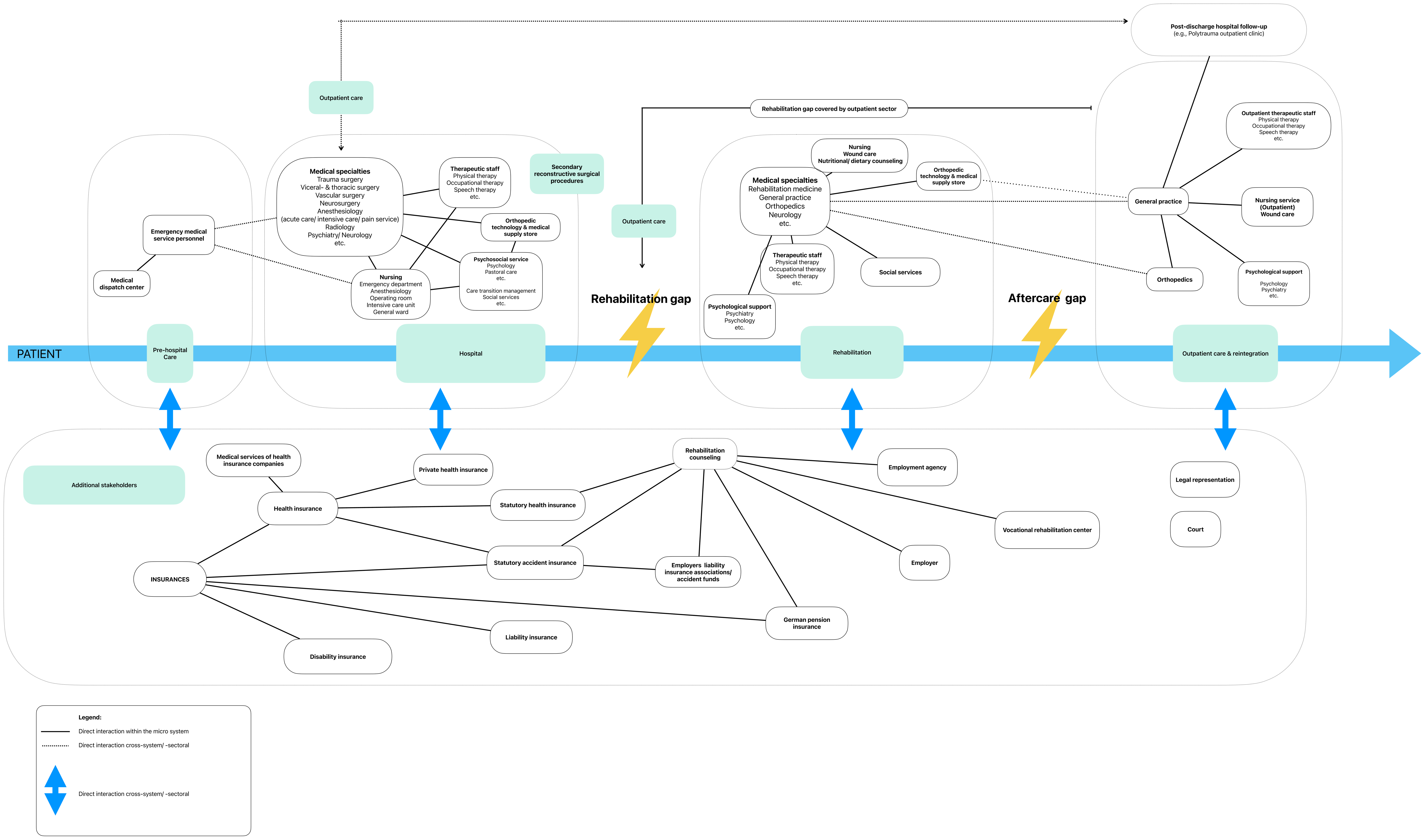

Post-discharge hospital follow-up
(e.g., Polytrauma outpatient clinic)
Outpatient care
Rehabilitation gap covered by outpatient sector
Secondary reconstructive surgical procedures
Medical specialties
Trauma surgery
Viceral- & thoracic surgery
Vascular surgery
Neurosurgery
Anesthesiology
(acute care/ intensive care/ pain service)
Radiology
Psychiatry/ Neurology
etc.
Therapeutic staff
Physical therapy
Occupational therapy
Speech therapy
etc.
Orthopedic technology & medical supply store
Psychosocial service
Psychology
Pastoral care
etc.
Care transition management
Social services
etc.
Nursing
Emergency department
Anesthesiology
Operating room
Intensive care unit
General ward
Emergency medical service personnel
Medical dispatch center
Outpatient care
Rehabilitation gap
Nursing
Wound care
Nutritional/ dietary counseling
Medical specialties
Rehabilitation medicine
General practice
Orthopedics
Neurology
etc.
Orthopedic technology & medical supply store
Therapeutic staff
Physical therapy
Occupational therapy
Speech therapy
etc.
Social services
Psychological support
Psychiatry
Psychology
etc.
Aftercare gap
Outpatient therapeutic staff
Physical therapy
Occupational therapy
Speech therapy
etc.
General practice
Nursing service (Outpatient)
Wound care
Orthopedics
Psychological support
Psychology
Psychiatry
etc.
PATIENT
Pre-hospital Care
Hospital
Rehabilitation
Outpatient care & reintegration
Additional stakeholders
Medical services of health insurance companies
Private health insurance
Health insurance
Statutory health insurance
Rehabilitation counseling
Employment agency
Vocational rehabilitation center
Employer
Legal representation
Court
INSURANCES
Statutory accident insurance
Employers liability insurance associations/ accident funds
German pension insurance
Liability insurance
Disability insurance
Legend:
Direct interaction within the micro system
Direct interaction cross-system/ -sectoral
Direct interaction cross-system/ -sectoral
